# Damage Evolution of Steel Fibre-Reinforced High-Performance Concrete in Low-Cycle Flexural Fatigue: Numerical Modeling and Experimental Validation

**DOI:** 10.3390/ma15031179

**Published:** 2022-02-03

**Authors:** Gregor Gebuhr, Mangesh Pise, Steffen Anders, Dominik Brands, Jörg Schröder

**Affiliations:** 1 Institute for Structural Engineering, Faculty of Architecture and Civil Engineering, Bergische Universität Wuppertal, Pauluskirchstraße 11, 42285 Wuppertal, Germany; s.anders@uni-wuppertal.de; 2 Institute of Mechanics, Department Civil Engineering, Faculty of Engineering, University of Duisburg-Essen, Universitätsstraße 15, 45141 Essen, Germany; dominik.brands@uni-due.de (D.B.); j.schroeder@uni-due.de (J.S.)

**Keywords:** high-performance concrete (HPC), steel-fibre reinforcement, low-cycle fatigue deterioration indicators, AE-analysis, phenomenological material model, elastoplastic phase-field model, three-point bending beam test, degradation of residual stiffness

## Abstract

This contribution aims to analyze the deterioration behaviour of steel fibre-reinforced high-performance concrete (HPC) in both experiments as well as numerical simulations. For this purpose, flexural tensile tests are carried out on beams with different fibre contents and suitable damage indicators are established to describe and calibrate the damage behaviour numerically using a phase-field model approach. In addition to conventional measurement methods, the tests are equipped with acoustic emission sensors in order to obtain a more precise picture of crack evolution by observing acoustic events. It is shown that, in addition to classical damage indicators, such as stiffness degradation and absorbed energy, various acoustic indicators, such as the acoustic energy of individual crack events, can also provide information about the damage progress. For the efficient numerical analysis of the overall material behaviour of fibre-reinforced HPC, a phenomenological material model is developed. The data obtained in the experiments are used to calibrate and validate the numerical model for the simulation of three-point bending beam tests. To verify the efficiency of the presented numerical model, the numerical results are compared with the experimental data, e.g., load-CMOD curves and the degradation of residual stiffness.

## 1. Introduction

Steel fibre-reinforced concrete (SFRC), high-performance concrete (HPC) with steel fibres, as well as ultra-high-performance concrete (UHPC) with fibres have been objects of research worldwide and have been widely applied throughout the last decades [1,2]. The advantages of steel fibres as reinforcement in concrete include increasing the load-bearing capacity of cracked concrete, reducing crack width to increase durability, and increasing energy absorption capacity and ductility under cyclic loading. Therefore, steel fibre-reinforced concrete has been applied in several areas. For example, in precast segments for tunnel linings, e.g., for the Brightwater tunnels or the Crossrail project in London [3,4,5]. Meanwhile, a state-of-the-art report for using steel fibres in precast tunnel segments has been published [6]. Another application of SFRC are joints in reinforced beam–column structures, which may be subjected to earthquakes [7,8,9,10]. In this case, steel fibres are added to improve the energy dissipation of the joints.

The load-bearing capacity and crack width-reduction capability of SFRC is also used for slabs on ground, such as industrial floorings, in transportation infrastructure [11,12], or in applications for field-cast ultra-high-performance concrete joints [13,14] as well as SFRC for concrete bridge decks or bridge deck overlays [13,15,16,17,18]. In the latter applications, additional cyclic loadings have to be considered. High-cycle loadings exceeding $10^{7}$ load cycles are expected, for example, for the support structures of offshore wind energy turbines or high-performance materials for grouting joints of precast segments of wind towers. Here, especially ultra-high-performance concrete with and without fibres is often used in the so called grouted connections [19,20].

In addition, the increasing compressive strength of HPC and UHPC is expected to decrease the dead weight of concrete structures, increasing their susceptibility to dynamic loads. Therefore, the performance of SFRC in terms of cyclic behaviour subjected to very high numbers of load cycles has to be closely investigated. Since the properties of SFRC differ depending on geometry, tensile strength, aspect ratio and amount of steel fibres, it is not possible to test every single concrete mix with respect to its high-cycle performance.

In order to gain deeper understanding of the composite material SFRC under high-cyclic loading, the German Science Foundation (DFG) has launched a comprehensive priority program (SPP2020). The project described here is part of this priority program. The overall goal of this project is to develop an efficient experimental–numerical approach that will allow researchers and engineers to combine short-term tests, such as low-cycle flexural fatigue tests, with numerical models to evaluate the performance of high-performance SFRCs under high-cycle loading without the need for time-consuming high-cycle fatigue tests. To calibrate the numerical models, the evolution of damage in the SFRC has to be known. Therefore, different damage indicators such as the evolution of consumed energy per load cycle, the evolution of stiffness or the evolution of acoustic emission events are described, which can be used to calibrate damage functions in the numerical model. A comparable approach was described by [21] for polymer fibre-reinforced concrete and by de Smedt [22] and Boulekbache et al. [23] for normal-strength fibre-reinforced concrete. In the former two contributions, a sectional analysis approach was applied as numerical models.

This paper describes specific, low-cycle, flexural tests on steel fibre-reinforced concrete with three different amounts of steel fibres, the development of various damage indicators including acoustic emission, and a numerical phase-field approach to fracture. Finally, the ability of the model to calculate the load crack-opening behaviour and the damage evolution thereof is shown.

The phase-field approach for fracture has recently become popular because of its prediction capability for complex crack propagation, see [24,25,26,27,28]. The works on the phase-field models for ductile fracture (see [29,30,31,32,33]) are followed for the development of an elastoplastic phase-field model. In our recent work using the small-strain elastoplastic phase-field model for fractures, see [34,35,36], the single steel-fibre pullout tests are elaborated experimentally and numerically to set up a framework for the study of fibre’s influence on the overall material behaviour of HPC. In this contribution the formulations presented in [37] are further developed to capture the numerical response of three-point bending beam tests for steel fibre-reinforced HPC.

This paper, as well as the underlying research effort, is divided into two main areas. Within the scope of the first area, the material behaviour is investigated experimentally with the aim of determining suitable indicators to describe damage development. For this purpose, as well as for calibrating and validating the numerical model, these indicators are needed to describe the development of the deterioration in the post-cracking phase [38,39,40,41,42,43]. For the determination of these damage indicators as well as for characterizing low- and high-cycle fatigue behaviour, unloading–reloading cycles are necessary. The second area is focused on the development of a phenomenological model for the prediction of the complex elastoplastic material behaviour of steel fibre-reinforced HPC under fatigue.

## 2. Materials and Experimental Methods

### 2.1. Concrete Mixtures and Fibres

All tests described here were performed on high-performance concrete bending beams of dimension of 700 × 150 × 150 mm${}^{3}$. The concrete composition used was developed as a reference mixture within the German priority programme SPP 2020; the exact component breakdown can be found in Table 1. As listed there, it is based on a Portland cement of type CEM I 52.5R. As aggregate, quartz sand is used in grain fractions from 0 to 2 mm and basalt gravel for those from 2 to 8 mm in grain size. Furthermore, a superplasticizer and stabilizer are added for better fresh concrete workability. The w–c ratio of the mixture is 0.352. In addition to the tests described in this contribution, comparable sets of tests are performed using ultra-high-performance concrete (UHPC) with high-strength, short, steel fibres.

The mechanical properties of the concrete mix shown in Table 2 were determined on standard cylindrical specimens. Stripping was performed one day after casting and testing started 28 days after production. All specimens were stored in water until testing.

The experiments were carried out on specimens containing 0/23/57/115 kg/m${}^{3}$ hook-end macro steel fibres of type 3D 65/60 with a tensile strength of 1150 MPa, kindly provided by Bekaert. These correspond to contents of 0/0.3/0.75/1.5% by volume, respectively. A total of 12 bending beam specimens are investigated in the test series described here, with three specimens per examined fibre set.

### 2.2. Experimental Setup and Loading

**Figure 1:**
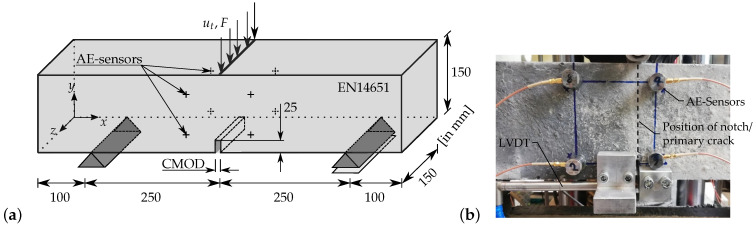
Three-point bending beam test: (**a**) geometry for the experimental setup and boundary value problem and (**b**) detailed photo of setup.

All specimens have been stored under water for 28 days until testing. In order to investigate damage development and, at the same time, to ensure a basic comparability between tests, three-point bending tests are carried out on notched specimens according to EN 14 651, with additional periodic unloading–reloading cycles. The test setup is shown in Figure 1.

In the tests accompanied by acoustic emission (AE) recording, piezoelectric AE-sensors type VS150-K by Vallen Systeme are attached to both sides of the beam along the longitudinal axis, in two planes containing four sensors each. The first plane is intended to provide the most accurate two-dimensional resolution possible of the primary crack propagation over a short distance of 20 mm from the notch of the beam. The second plane of sensors is intended to quantify the size of the three-dimensional crack process zone over a longer distance of 100 mm to the other side of the notch. The sensors are connected to a Vallen AMSY-6 system through pre-amplifiers with a gain level of 34 dB. The most relevant calibration settings for the system are summarized in Table 3.

As mentioned earlier, the experiments are performed with periodic unloading–reloading cycles. The cycles at the beginning of the test (CMOD values of up to 0.05mm) are carried out at crack-opening increments of 0.01 mm in order to make the crack initiation traceable as precisely as possible. For larger crack openings, the individual crack opening target value is doubled for each, following the unloading cycle, namely CMOD values of 0.1/0.2/0.4/…/3.2 mm. All tests were stopped at a CMOD of 4.0 mm. All phases with an increasing crack mouth opening displacement CMOD (loading phases) were controlled using the CMOD. All phases with a decreasing CMOD (unloading phases) were force-controlled in order to be able stop at a given lower load limit automatically.

### 2.3. Damage Indicators

The aim of this contribution is to experimentally quantify and numerically simulate not only the envelope of the load-CMOD curve but also to quantify the ongoing deterioration within the fibre-modified high-performance concrete. For this purpose, different damage indicators can be derived from the experiments. As stated above, unloading curves are necessary to differentiate between elastic crack openings, plastic crack openings and to further determine residual stiffnesses, especially after the occurrence of the first crack. In the literature [22,23,39,41,42,43] different indicators are proposed to quantify damage development throughout a low-cycle or high-cycle fatigue test. Mechanical indicators that are often mentioned are the plastic part of the crack-opening, the residual stiffness (sometimes referred to as compliance) and the energy that is consumed to open the crack. With regard to the damage-effective energy, different approaches must be distinguished. For example, in static tests, an integral over the load-CMOD curve can be used to determine the energy consumed [39,41]. For force-controlled, high-cycle fatigue tests, the damage energy can be interpreted as the portions of the load-CMOD curve enclosed by the hysteresis loops, as shown in Bode et al. [46]. A second approach for such tests is to interpret the trapezoid enclosed by the upper and lower load levels and initial and residual stiffness as the damage energy, an approach taken, for example, in Gebuhr et al. [41], De Smedt et al. [43]. The latter approach is transferable to fatigue tests with a low number of load cycles, such as those investigated in this paper, since residual stiffnesses can also be determined here, thus enabling better comparability with high-cycle fatigue tests.

**Figure 2:**
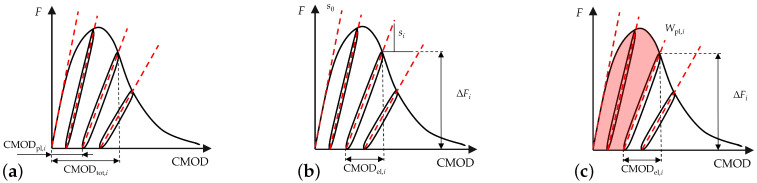
Sketches for the damage indicators in quasi-static tests: (**a**) CMOD plastic (CMOD${}_{\mathrm{pl},i}$), (**b**) initial ($s_{0}$) and residual ($s_{i}$) stiffness and (**c**) damage energy ($W_{\mathrm{pl},i}$).

In this contribution, three damage indicators are derived and displayed from the experiments and subsequently used in the numerical model. In Figure 2 these indicators are sketched.

For all indicators, a reference frame has to be set. In this contribution, the total CMOD for each unloading cycle (CMOD${}_{\mathrm{tot},i}$) is applied, defined as the intersection of the unloading and consecutive reloading branch of the load-CMOD curve of each individual load cycle. The CMOD${}_{\mathrm{tot}}$ at the beginning of each unloading cycle is defined according to ${\mathrm{CMOD}}_{\mathrm{tot},i}={\mathrm{CMOD}}_{\mathrm{pl},i}+{\mathrm{CMOD}}_{\mathrm{el},i}$, whereby the plastic ${\mathrm{CMOD}}_{\mathrm{pl},i}$ is additionally used as a damage indicator. Another well-known damage indicator is the gradient module of the $i^{th}$ unloading–reloading cycle, the residual stiffness $s_{i}$, calculated as

(1) $$s_{i}=\frac{\Delta F_{i}}{{\mathrm{CMOD}}_{\mathrm{tot},i}-{\mathrm{CMOD}}_{\mathrm{pl},i}}\;,$$

where ΔF describes the load difference between the CMOD${}_{\mathrm{tot},i}$- and CMOD${}_{\mathrm{pl},i}$-assigned points of the curve. In a second step this stiffness can be transformed to a dimensionless damage value, $q_{i}$, as used in the numerical model, by normalizing it using the initial stiffness $s_{0}$, i.e.,

(2) $$q_{i}=\frac{s_{0}-s_{i}}{s_{0}}\;.$$

The dimensionless damage parameter $q_{i}$ is then used for the calibration of the numerical model to formulate the degradation function $g(q_{i})$. The last indicator is the plastic or damage energy, $W_{\mathrm{pl},i}$. It is defined as the integral of the load-CMOD curve up to CMOD${}_{\mathrm{tot},i}$; reduced by the elastic energy at this point, it reads

(3) $$W_{\mathrm{pl},i}=\int_{0}^{{\mathrm{CMOD}}_{\mathrm{tot},i}}F\;d\mathrm{CMOD}-\frac{{\mathrm{CMOD}}_{\mathrm{el},i}*F_{\max,i}}{2}\;.$$

For the calibration of the numerical model of the load-CMOD curve, plastic crack opening CMOD${}_{\mathrm{pl},i}$ and the residual stiffness are of primary importance, as the calculation of the plastic energy is based on them. The challenge for the numerical model is to correctly predict not only the named indicators but also their development with an increasing fibre content.

**Figure 3:**
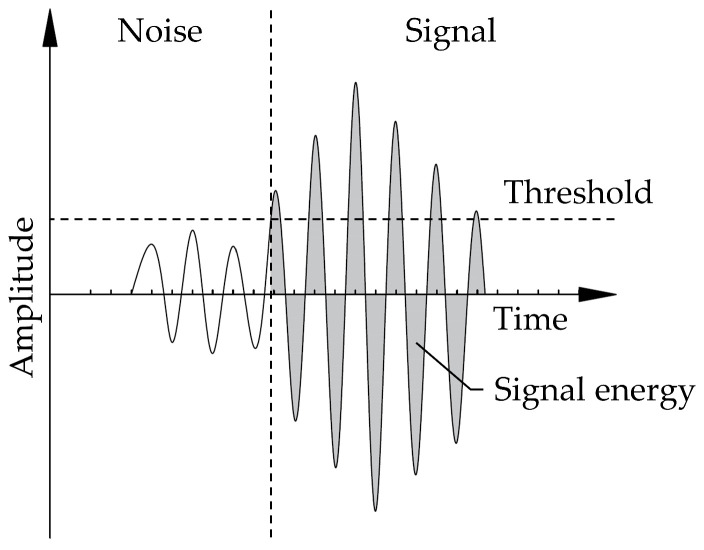
Schematic waveform of a signal.

Apart from the aforementioned mechanical damage indicators, an analysis of the acoustic phenomena can also be used to assess and describe the damage process. The analysis of the data is divided into different levels, all of which revolve around the analysis of the so called hits, i.e., the signal shape after a predefined threshold value has been exceeded. The first level considered here is the direct evaluation of the time-domain characteristics for hits recorded by the individual microphones, such as the maximum amplitude and overall energy of the hit, as depicted in Figure 3. These values can be used to determine the general activity in the test specimen, as shown e.g., in De Smedt et al. [43], Nguyen [47].

### 2.4. Numerical Model

For the numerical solution, we used the small-strain, elastoplastic phase-field model for fracture in reinforced high-performance concrete, see Pise et al. [35], Storm et al. [36], Pise et al. [37]. In the context of small strain, the displacement field is u and the phase-field parameter is $q\in \left[0,1\right]$, which serves as a damage parameter, see Miehe et al. [25]. An additive structure of the macroscopic stored energy function per unit volume ψ is introduced as

(4) $$\psi =v^{\mathrm{HPC}}\;\psi^{\mathrm{HPC}}\left(\varepsilon ,\varepsilon^{p,\mathrm{HPC}},q,\nabla q,\alpha^{\mathrm{HPC}}\right)+v^{F}\;\psi^{F}\left(\varepsilon ,M,e^{p,F},\alpha^{F}\right)\;\mathrm{with}\;\varepsilon^{e,\mathrm{HPC}}:=\varepsilon -\varepsilon^{p,\mathrm{HPC}}\;,$$

where the variables $\alpha^{\mathrm{HPC}}$ and $\alpha^{F}$ are the equivalent plastic strains in the HPC phase and in the fibre phase, respectively. Elastic $\varepsilon^{e,\mathrm{HPC}}$ and plastic $\varepsilon^{p,\mathrm{HPC}}$ strains in the HPC phase are the parts of the total strain tensor ε that can be expressed using the symmetric displacement gradient, $\nabla_{s}u$, as $\varepsilon =\nabla_{s}u=\frac{1}{2}\left(\nabla u+\nabla^{T}u\right)$. It is observed in the experiments that during the failure of steel fibre-reinforced HPC the fracture mainly occurs only in the HPC phase. On that account, the phase-field parameter q∈[0,1] is considered only in the HPC phase, representing the damage therein. Its gradient is denoted by ∇q. The symbols $v^{\mathrm{HPC}}$ and $v^{F}$ describe the volume fraction of the HPC phase and fibre phase, respectively. The are conserved by the constraint $v^{\mathrm{HPC}}$ = $1-v^{F}$.

The energy function $\psi^{\mathrm{HPC}}$ describes the mechanical behaviour of the HPC phase, cf. [32], reads

(5) $$\psi^{\mathrm{HPC}}=g\left(q\right)\left[\psi_{0}^{e+,\mathrm{HPC}}+\psi_{0}^{p,\mathrm{HPC}}-\psi^{c,\mathrm{HPC}}\right]+\psi_{0}^{e-,\mathrm{HPC}}+\psi^{c,\mathrm{HPC}}+2\;\frac{\psi^{c}}{\zeta }\;l\left[\frac{1}{2l}\;q^{2}+\frac{l}{2}{\;||\nabla q||}^{2}\right]\;.$$

The specific critical fracture energy $\psi^{c,\mathrm{HPC}}>0$ controls the crack threshold and the vanishing length scale parameter l→0 achieves a sharp crack interface. The parameter ζ controls the post-critical stress softening due to the evolution of the fracture and $g\left(q\right)$, denoting a degradation function, depends on the phase-field parameter *q*. A positive part $\psi_{0}^{e+,\mathrm{HPC}}\left(\varepsilon^{e,\mathrm{HPC}}\right)$ and a negative part $\psi_{0}^{e-,\mathrm{HPC}}\left(\varepsilon^{e,\mathrm{HPC}}\right)$ of the reference elastic energy function $\psi_{0}^{e,\mathrm{HPC}}\left(\varepsilon^{e,\mathrm{HPC}}\right)$ as proposed in Amor et al. [48], read

(6) $$\psi_{0}^{e+,\mathrm{HPC}}\left(\varepsilon^{e,\mathrm{HPC}}\right)=\kappa {\langle \mathrm{tr}\left[\varepsilon^{e,\mathrm{HPC}}\right]\rangle }_{+}^{2}/2+\mu {\left|\right|\mathrm{dev}\varepsilon^{e,\mathrm{HPC}}\left|\right|}^{2}\;\mathrm{and}\;\psi_{0}^{e-,\mathrm{HPC}}\left(\varepsilon^{e,\mathrm{HPC}}\right)=\kappa {\langle \mathrm{tr}\left[\varepsilon^{e,\mathrm{HPC}}\right]\rangle }_{-}^{2}/2\;.$$

This formulation ensures that the evolution of damage only occurs due to the volume expansion of material. Therein, μ and κ are the Lam$\overset{´}{e}$ coefficients of the HPC phase and Macaulay’s notation describes the operation ${\langle •\rangle }_{\pm }=1/2\;\left(\;•\pm |•|\;\right)$. The considered plastic energy, $\psi^{p,\mathrm{HPC}}$, reads

(7) $$\psi^{p,\mathrm{HPC}}\left(\alpha^{\mathrm{HPC}}\right)=g\left(q\right)\psi_{0}^{p,\mathrm{HPC}}\;\mathrm{with}\;\psi_{0}^{p,\mathrm{HPC}}=\left[y_{0}^{\mathrm{HPC}}\;\alpha^{\mathrm{HPC}}+\frac{1}{2}\;h^{\mathrm{HPC}}\;{\left(\alpha^{\mathrm{HPC}}\right)}^{2}\right]\;,$$

which depends on the equivalent plastic strain $\alpha^{\mathrm{HPC}}$, yield stress $y_{0}^{\mathrm{HPC}}$ and hardening parameter $h^{\mathrm{HPC}}$ for the HPC phase, respectively.

The energy function for the fibre phase $\psi^{F}$ characterizing the embedded steel fibre in a preferred fibre direction a with ||a||=1 reads

(8) $$\psi^{F}\left(\varepsilon ,M,e^{p,F},\alpha^{F}\right)=\psi^{e,F}\left(\varepsilon ,M,e^{p,F}\right)+\psi^{p,F}\left(\alpha^{F}\right)\;\mathrm{where}\;e^{e,F}=e^{F}-e^{p,F}\;\mathrm{with}\;e^{F}=\varepsilon :M\;,$$

where $\alpha^{F}$ is the equivalent plastic strain for the fibre phase. The total strain tensor $e^{F}$ of the fibre phase along the preferred fibre directions can be decomposed into elastic $e^{e,F}$ and plastic $e^{p,F}$ parts. Structural tensor M can be formulated using the preferred fibre direction a as M:=a⊗a, cf. [49]. With this the one-dimensional elastoplasticity problem representing the three-dimensional elastoplasticity for the embedded steel fibre, oriented in a preferred fibre direction, a is formulated. Therein, an elastic $\psi^{e,F}$ and a plastic $\psi^{p,F}$ energy function are considered as

(9) $$\psi^{e,F}\left(\varepsilon ,M,e^{p,F}\right)=\frac{1}{2}\;E^{F}{\left(e^{F}-e^{p,F}\right)}^{2}\;\mathrm{and}\;\psi^{p,F}\left(\alpha^{F}\right)=y_{0}^{F}\;\alpha^{F}+\frac{1}{2}\;h^{F}\;{\left(\alpha^{F}\right)}^{2}\;,$$

where $E^{F}$, $y_{0}^{F}$ and $h^{F}$ are the elastic moduli, initial yield stress and hardening parameter for the fibre phase, respectively. The stress tensor additively, composed by the stress tensor $\sigma^{\mathrm{HPC}}$ for the HPC phase and $\sigma^{F}$ for the fibre, phase reads

(10) $$\sigma :=v^{\mathrm{HPC}}\;\sigma^{\mathrm{HPC}}+v^{F}\;\sigma^{F}\;.$$

The stress tensor $\sigma^{F}$ for the fibre phase is defined as

(11) $$\sigma^{F}:=\sigma^{F}a\;⊗\;a\;\mathrm{with}\;\sigma^{F}=\partial_{e^{e,F}}\psi^{F}=E^{F}\left(e^{F}-e^{p,F}\right).$$

A stress tensor for the HPC phase $\sigma^{\mathrm{HPC}}$ can be obtained by

(12) $$\sigma^{\mathrm{HPC}}:=\partial_{\varepsilon^{e,\mathrm{HPC}}}\psi^{\mathrm{HPC}}=g\left(q\right)\underset{\sigma_{0}^{+,\mathrm{HPC}}}{\underset{︸}{\left[\kappa^{\mathrm{HPC}}{\langle \mathrm{tr}\;\varepsilon^{e,\mathrm{HPC}}\rangle }_{+}I+2\mu^{\mathrm{HPC}}\;\mathrm{dev}\varepsilon^{e,\mathrm{HPC}}\right]}}+\underset{\sigma_{0}^{-,\mathrm{HPC}}}{\underset{︸}{\left[\kappa^{\mathrm{HPC}}{\langle \mathrm{tr}\;\varepsilon^{e,\mathrm{HPC}}\rangle }_{-}I\right]}}\;,$$

where $\sigma_{0}^{+,\mathrm{HPC}}$ and $\sigma_{0}^{+,\mathrm{HPC}}$ are the positive and the negative part of the effective stress tensor $\sigma_{0}^{\mathrm{HPC}}$ for the HPC phase. Here, the degradation function $g\left(q\right)$ is a data-driven function that depends on the evolution of the phase-field parameter *q*. Two different data sets are considered for the degradation function, $g^{+}\left(q\right)$, in tension, and $g^{-}\left(q\right)$, in compression. These degradation functions are calibrated using the experimental data for the three-point bending beam test for pure HPC at low cycle. For the computation of the governing equation for the phase-field parameter *q*, the degradation function $g\left(q\right)={\left(1-q\right)}^{2}$ is considered. It is calculated from the derivative of the macroscopic stored energy function ψ with respect to the phase-field parameter *q*, which results in

(13) $$q-l^{2}\;\mathrm{Div}\left(\nabla q\right)-\left(1-q\right)H^{\mathrm{HPC}}=0\;,$$

where the maximum value of the local history field H, which ensures the irreversibility of the evolution of damage, is given by

(14) $$H^{\mathrm{HPC}}:=\max_{s\in [0,t]}\zeta \langle \frac{v^{\mathrm{HPC}}\left[\psi_{0}^{e+,\mathrm{HPC}}+\psi_{0}^{p,\mathrm{HPC}}\right]}{\psi^{c,\mathrm{HPC}}}-1\rangle .$$

The unique behaviour of concrete in tension and in compression is captured by using different parameters for the critical fracture energy in tension $\psi_{t}^{c,\mathrm{HPC}}$ and in compression $\psi_{c}^{c,\mathrm{HPC}},$ for details see Storm et al. [36]. They are differentiated from each other by the sign of the first invariant of the stress tensor $\mathrm{tr}\;\sigma^{\mathrm{HPC}}$ for the HPC phase. Two different yield criteria are considered for the description of non-linear behaviour of the HPC and fibre phases. This gives the flexibility to use different yield criteria to predict the elastoplastic behaviour of the reinforcement, as well as of the concrete materials. The non-associative Drucker–Prager yield criteria is used for the HPC phase, which is capable of capturing the asymmetric tension–compression property of concrete. A potential function of Drucker–Prager yield criteria, $\phi_{p}^{\mathrm{HPC}}$, is defined by cf. [50,51],

(15) $$\phi_{p}^{\mathrm{HPC}}=\frac{1}{\sqrt{2}}\;||\mathrm{dev}\sigma_{0}^{\mathrm{HPC}}\left|\right|-\beta_{p}\mathrm{tr}\;\sigma_{0}^{\mathrm{HPC}}-\kappa_{p}^{\mathrm{HPC}}\;\mathrm{with}\;\kappa_{p}^{\mathrm{HPC}}:=\partial_{\alpha^{\mathrm{HPC}}}\psi_{0}^{p,\mathrm{HPC}}=y_{0}^{\mathrm{HPC}}+h^{\mathrm{HPC}}\alpha^{\mathrm{HPC}}\;,$$

and the plastic potential function, $\phi_{n}^{\mathrm{HPC}}$, is considered as

(16) $$\phi_{n}^{\mathrm{HPC}}=\frac{1}{\sqrt{2}}\;||\mathrm{dev}\sigma_{0}^{\mathrm{HPC}}\left|\right|-\beta_{n}\mathrm{tr}\;\sigma_{0}^{\mathrm{HPC}}\;,$$

where $\beta_{p}$ and $\beta_{n}$ are the material parameters. Note that these potentials are the functions of the effective stress tensor $\sigma_{0}^{\mathrm{HPC}}$ for the HPC phase. The equation describing the evolution of plastic strains ${\dot{\varepsilon }}^{p,\mathrm{HPC}}$, equivalent plastic strains ${\dot{\alpha }}^{\mathrm{HPC}}$ and expressed in terms of the incremental plastic consistency parameter $\lambda^{p,\mathrm{HPC}}$, and the Kuhn–Tucker conditions for the HPC phase read

(17) $${\dot{\varepsilon }}^{p,\mathrm{HPC}}=\lambda^{p,\mathrm{HPC}}\;\frac{\partial \phi_{n}^{\mathrm{HPC}}}{\partial \sigma_{0}^{\mathrm{HPC}}}\;,\;{\dot{\alpha }}^{\mathrm{HPC}}=\lambda^{p,\mathrm{HPC}}\;\mathrm{and}\;\phi_{p}^{\mathrm{HPC}}\leq 0\;,\;\;\lambda^{p,\mathrm{HPC}}\geq 0\;,\;\;\lambda^{p,\mathrm{HPC}}\phi_{p}^{\mathrm{HPC}}=0\;.$$

To describe the non-linear behaviour along the preferred fibre direction, the one-dimensional von Mises yield criterion is used, i.e.,

(18) $$\phi^{F}\left(\sigma^{F},\kappa_{p}^{F}\right)=\left|\sigma^{F}\right|-\kappa_{p}^{F}\;\mathrm{with}\;\kappa_{p}^{F}:=\partial_{\alpha^{F}}\psi^{p,F}=y_{0}^{F}+h^{F}\alpha^{F}\;.$$

The evolution equation of the plastic strain ${\dot{e}}^{p,F}$ and the equivalent plastic strain $\alpha^{F}$ using Kuhn–Tucker conditions to describe the loading and unloading conditions for the fibre phase are

(19) $${\dot{e}}^{p,F}=\lambda^{p,F}\;\frac{\partial \phi^{F}}{\partial \sigma^{F}}\;,\;{\dot{\alpha }}^{F}=\lambda^{p,F}\;\mathrm{and}\;\phi^{F}\leq 0\;,\;\;\lambda^{p,F}\geq 0\;,\;\;\lambda^{p,F}\phi^{F}=0\;,$$

where $\lambda^{p,F}$ denotes the incremental plastic consistency parameter for the fibre phase. The system of equations contains the balance of linear momentum using Equation (10) and the governing equation for the phase-field parameter, i.e., Equation (13), in domain B along with boundary conditions σ·n=t and ∇q·n=0 on the surface of the domain ∂B. For the analysis of failure in reinforced HPC, the presented numerical model is implemented in the framework of the finite element method and solved using the incrementally decoupled updates using a staggered scheme, cf. [25,30].

## 3. Experimental and Numerical Results

### 3.1. Load-CMOD Curves

**Figure 4:**
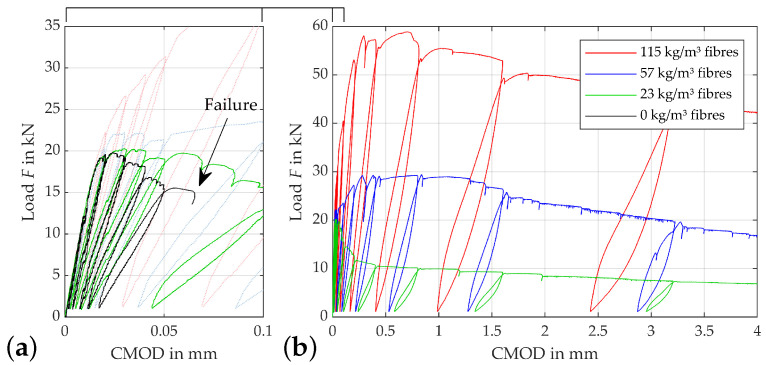
Exemplary load-CMOD curves: (**a**) zoomed view of (**b**) plot for different fibre contents.

The load-CMOD curves displayed in Figure 4 show the effects of fibre reinforcement on the ductility and load-bearing capacity. All specimens tested show similar curves up to a CMOD of approx. 0.015 mm. From this point, plastic deformation starts, as the crack initiates. The mixture without fibres (black line) has only very limited ability to bridge this crack opening. Failure occurs nearly immediately after the maximum load has been reached. The mixture with a fibre content of 23 kg/m${}^{3}$ and 0.3 vol.-% (green line) shows typical subcritical behaviour. The forces transferred over the crack decrease continuously after the flexural strength is reached. The blue line represents the behaviour with a fibre content of 57 kg/m${}^{3}$ respectively 0.5 vol.-%. This line shows typical critical behaviour, which means that force transfer can be maintained by the fibres after the crack opening starts and the flexural strength is preserved, even at higher crack openings. In this curve it can also be seen that it is important to distinguish between the load that can be absorbed at first crack, which is reached when the force CMOD curve deviates from a linear elastic slope, and the maximum load that can be absorbed. In the case of strain-hardening behaviour as shown here, this force is much higher than the force at first crack, meaning that the high flexural strength is attributable to the high number of fibres and their load bearing capacity. This difference is more pronounced if the fibre content is increased to 115 kg/m${}^{3}$ or 1.5% by volume. Here, the first crack still occurs at about 25 kN, but flexural strength is reached at about 58 kN at CMOD${}_{\mathrm{tot},i}$ of about 0.6mm.

### 3.2. Mechanical Damage Indicators

**Figure 5:**
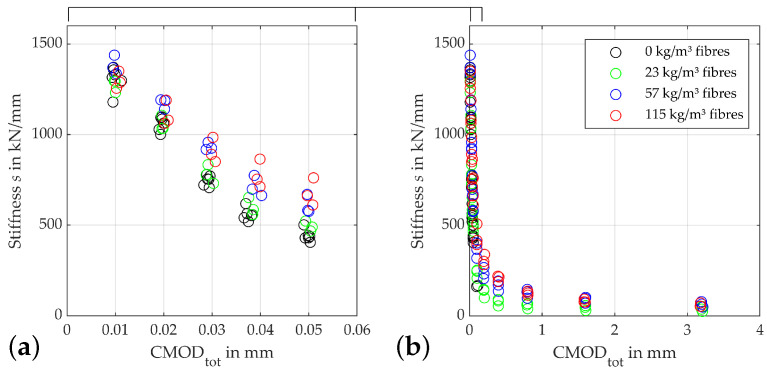
Development of residual stiffness: (**a**) zoomed view of (**b**) plot for different fibre contents.

As prior research has shown [39,41,43], one feasible indicator for the overall deterioration process is the evolution of the residual stiffnesses, i.e., the gradient modules of the individual load cycles. Figure 5 depicts the development of residual stiffnesses of concretes containing different fibre contents. To get a better resolution of the first unloading cycles up to a CMOD of 0.06 mm, which is the failure point of the fibre-free specimens, a detail view is added up for this CMOD, which will be given for all following figures depicting damage indicators.

Since it holds true for all beams that the first unloading cycle initiated at a CMOD${}_{\mathrm{tot}}$ of 0.01 mm is regraded as the linear elastic part of the load-CMOD curve, the first stiffness determined can be understood as the initial stiffness of the uncracked material. Since the fibres in the specimen are not activated at this point, all stiffnesses are close to each other, on average, at approx. 1300 kN/mm. As stated above, the specimens start to develop plastic deformations at a CMOD of approx. 0.015 mm. While the crack opening itself leads to a decrease in stiffness for all fibre contents, the steepness of this decrease is determined by the respective fibre content, with the fibre-free specimens degrading fastest. Furthermore, a higher fibre quantity seems to lead to a more pronounced scatter in the curve. Except for the fibre-free specimens, which fail at a crack-mouth opening of about 0.06 mm, the fibre content-dependent degradation rate continues up to crack openings of about 2 mm. From this point onward, the supercritical fibre contents of above 57 kg/m${}^{3}$ are, again, closer together, at a stiffness level of about 100 kN/mm. Beams with a subcritical fibre content of 23 kg/m${}^{3}$ achieve residual stiffnesses of about 75kN/mm at these crack openings.

**Figure 6:**
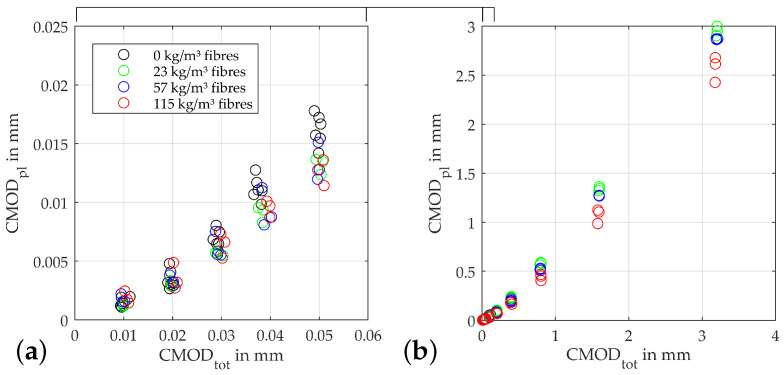
Plastic deformation after unloading: (**a**) zoomed view of (**b**) plot for different fibre contents.

The development of the plastic crack opening CMOD${}_{\mathrm{pl}}$ at the lower reversal point of each load cycle as shown in Figure 6 also indicates a clear influence of the fibre content on the behaviour of the specimens starting shortly after the initiation of the damage to the concrete matrix. This becomes evident from a CMOD${}_{\mathrm{tot}}$ of 0.03 mm, above which the fibre-free specimens begin to show significantly larger plastic crack openings than the beams with fibres. The plastic deformations of the remaining beams first start to scatter significantly more up to this value. From a CMOD${}_{\mathrm{tot}}$ of 0.4mm the specimens plastic crack openings start to order according to their fibre contents, with the beams with the $115\;\mathrm{kg}/m^{3}$ showing the smallest values. This trend increases in the rest of the test, up to 3.2 mm total crack opening, where these beams show an average CMOD${}_{\mathrm{pl}}$ of 2.6 mm. The beams with 23 kg/m${}^{3}$ fibres are at 2.99mm at this point, i.e., on average, with approx. at 12% larger plastic crack openings. The difference in these quantities is primarily due to the greater residual forces that can be transferred by the material as the fibre quantity increases. Since a convergence of the stiffnesses between the different fibre contents takes place with larger crack openings, the plastic deformation is correspondingly smaller. Overall, it is also noticeable that the plastic parts of the crack openings in the tests on the beams with a fibre content of $115\;\mathrm{kg}/m^{3}$ start to scatter more than with the lower fibre contents.

**Figure 7:**
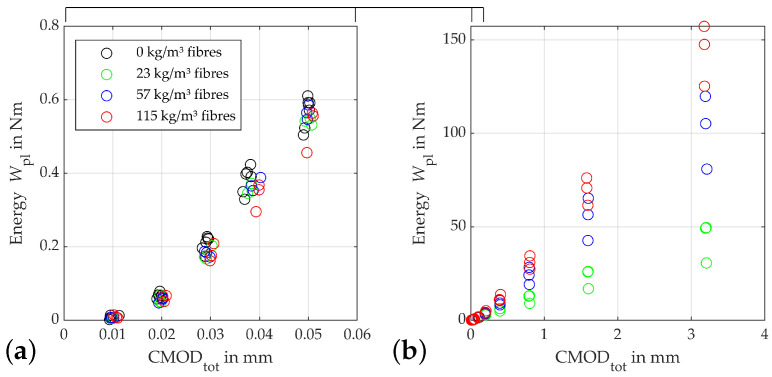
Absorbed damaging energy $W_{\mathrm{pl},i}$: (**a**) zoomed view of (**b**) plot for different fibre contents.

Figure 7 shows the progression of the absorbed damage energy. Here, the increasing absorption capacity of the beams with increasing fibre contents becomes clear. After the beams again show a behaviour, as with the aforementioned damage indicators, up to a crack opening of 0.3 mm—including the progressive fanning out into a wider dispersion of the calculated energies—from this point onwards, they again arrange themselves according to their fibre content. This process continues up to a crack opening of 0.8 mm. From this point on, the energy for an increase in crack opening rises approximately proportionally to the fibre content.

### 3.3. Acoustic Emission Measurements

**Figure 8:**
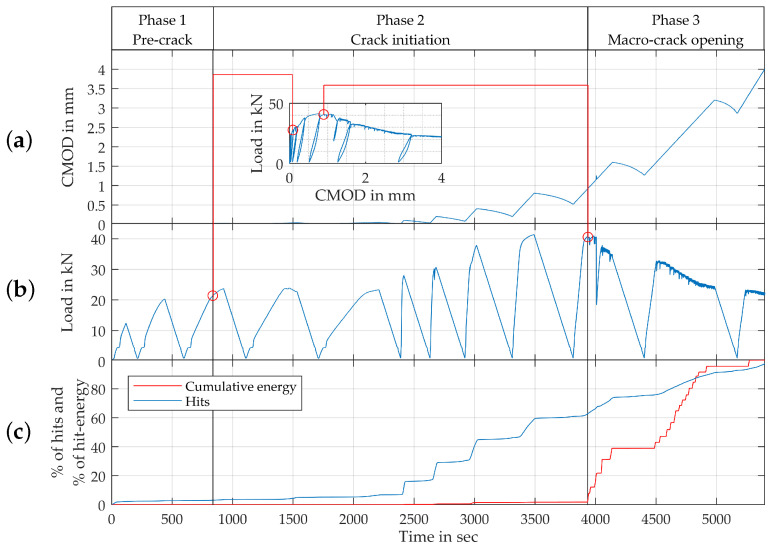
Phases of deterioration, progression of (**a**) CMOD, (**b**) load and (**c**) registered hits (total and weighted by respective energy) over time.

Figure 8 shows an exemplary experiment on a bending beam with 57 kg/m${}^{3}$ fibres, which corresponds to 0.75% by volume. Here, both the progression of CMOD as well as load are given over time. These progressions are now compared to the the data collected with the aid of the AE-sensors. Firstly, the AE-data is given via the relative number of raw hits detected after each point in time in relation to the total number of recorded hits over the course of the test. Secondly, the results are normalized using the respective energy of each of those hits to calculate cumulative energy.

The results are divided into distinct phases, starting with the pre-crack phase up to a CMOD of about 0.02 mm at about 800 s. Here, the crack initiation phase starts—mechanically defined by the loss of stiffness and beginning of plastic deformation, as described above. In the visualization above, this loss of stiffness can be seen in the gradient change of the residual force marked with a red circle. Following this point, micro-cracks begin to accumulate into a macro-crack, resulting into non-linearly increasing CMOD values. In this phase, the number of hits registered rises significantly. Interestingly, the beginning of the increase in total hits corresponds to the first significant increase in overall level of applied force after the beginning of the crack initiation phase. This effect however seems to have nearly no impact on the relative cumulative acoustic energy registered. While the absolute count of hits surpasses 60% of the total number of hits registered over the course of the experiment at the 3800 s mark of the test, the cumulative energy of the hits stays well below 5% of the total energy of hits registered by the microphones. The energy first starts to rise shortly after the maximum force is reached at a CMOD of approximately 0.8 mm, again labeled with a red circle in the curve. This point marks the beginning of a macro-crack-opening phase. As the acoustic events start to emit more energy, the overall load-bearing capacity of the beam degrades. This indicates a disparity between the first activation of the fibre and the minimum fibre pull-out necessary to mechanically activate the fibre.

### 3.4. Simulations of Three-Point Bending Tests at Low Cycle for Reinforced HPC

In this section, the simulation results of three-point bending beam tests at low cycle are reported. For these simulations, the mechanical properties of HPC are taken from the cumulative results of various partners within SPP 2020 program, for comparison, [34,44,45] (see Table 2). The material parameters and interpolated values of the data-driven degradation functions for tension $g^{+}\left(q\right)$ and for compression $g^{-}\left(q\right)$ are calibrated simultaneously for the uniaxial cyclic tension test, uniaxial cyclic compression test and three-point bending test for pure HPC at low cycle. The mechanical properties of HPC and the calibrated parameters used for the simulation are listed in Table 4.

The calibrated values for the interpolation of degradation functions for HPC in tension $g^{+}\left(q\right)$ and compression $g^{-}\left(q\right)$, with respect to corresponding values of the phase-field parameter, are listed in Table 5.

**Figure 9:**
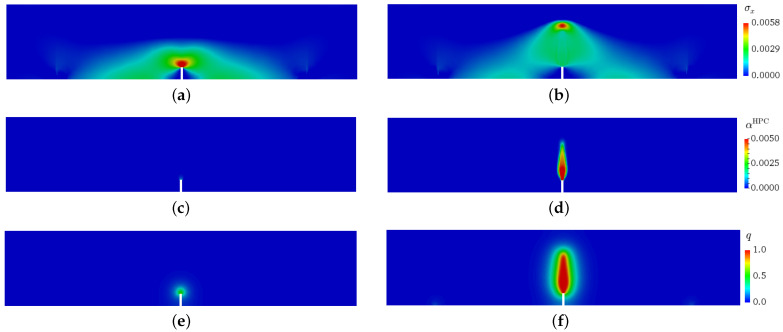
Three-point bending beam test at low cycle for fiber-reinforced HPC: distribution of stresses $\sigma_{x}$ (in GPa) in horizontal direction (*x*-axis), the equivalent plastic strain $\alpha^{\mathrm{HPC}}$ for HPC phase, the phase-field parameter *q* for HPC phase in (**a**,**c**,**e**) at CMOD = 0.018 mm and (**b**,**d**,**f**) at CMOD = 0.108 mm, respectively.

The geometry and the boundary conditions of the three-point bending beam test are shown in Figure 1a. All the dimensions shown in Figure 1a are taken as per the European Standard EN 14651. For all simulations, a beam of reinforced HPC with fibre content of 23 kg/mm${}^{2}$, i.e., 0.3 vol.-%, is considered. The three-dimensional state is considered by a plane stress approximation. Therefore the x-y-cross section of the beam is discretized by a finite element mesh with 3673 eight-noded hexahedral elements and unit-length thickness. A Maximum element size of 0.5 mm is taken for elements in the area surrounding the notch at the middle of the beam. As shown in Figure 1a, load is applied by a vertical displacement boundary condition, $u_{t}$, on the top surface of the beam, and the reaction forces of the constrained nodes at this tip are computed. To calculate the crack mouth-opening displacement (CMOD) the relative horizontal displacement of the opposite corners of the notch at the bottom of the beam is used, see Figure 1a. The distribution of stresses in the x-direction, $\sigma_{x}$ (in GPa), the equivalent, plastic strain. $\alpha^{\mathrm{HPC}}$, the phase-field parameter, *q*, at CMOD = 0.018 mm are shown in Figure 9a,c,e, and at CMOD = 0.108 mm in Figure 9b,d,f, respectively.

**Figure 10:**
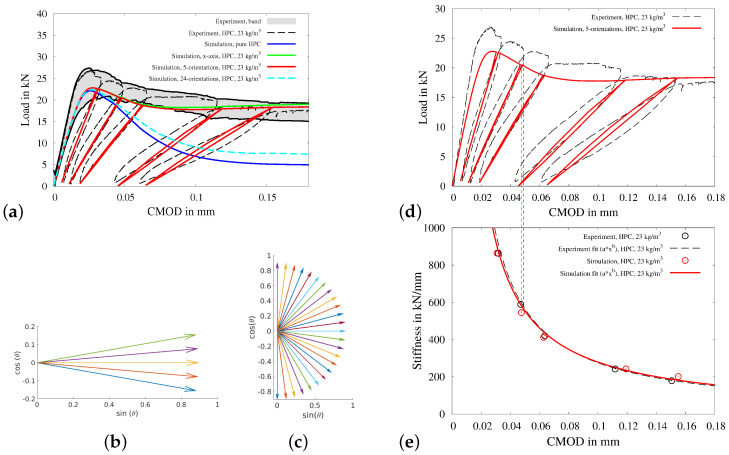
Three-point bending beam test for fiber-reinforced HPC: (**a**) load-CMOD diagramm for experimental data and simulations using (**b**) five preferred orientations between the angles of −10° to 10° and (**c**) 24 preferred orientations between the angles of −90° to 90°. Comparison of experimental and simulated (**d**) load-CMOD diagram and (**e**) calculated and interpolated residual stiffness-CMOD diagram.

In Figure 10a, the experimental and numerical load-CMOD curves are plotted. Therein, a scatter band of the experimental data and a representative experimental curve are shown in the gray area and by the black dotted curve, respectively. The numerical load-CMOD curve for pure HPC—the blue curve in Figure 10a—and reinforced HPC for a preferred fibre direction along x-axis—green curve in Figure 10a— represent the lower and upper bound for the load-CMOD curves for different combinations of preferred fiber direction.

The load-CMOD curve using five preferred fibre orientations—red curve in Figure 10a— between the angles of −10° to 10° (see Figure 10b) and using 24 preferred fibre orientations—cyan curve in Figure 10a—between the angles of −90° to 90° (see Figure 10c) are plotted in Figure 10a. For the analysis, load-CMOD curves for the experiment and for the simulation using five preferred fibre orientations are compared in Figure 10d. The development of the residual stiffness of the beam for simulation using five preferred fibre orientations is calculated for the each loading cycle, using the procedure explained in Figure 2b. For the calculation, the value of load at midpoint of the unloading and consequent reloading branch is calculated (see top point of dotted red vertical line in Figure 10d). The calculated residual stiffness-CMOD curves for the experiment and for the simulation using five preferred fibre orientations are plotted in the black and red circles, respectively, in Figure 10e. These calculated values of residual stiffness are used to get the interpolated residual stiffness-CMOD experimental, i.e., dotted black curve, and numerical curves, i.e., solid red curve. These interpolated residual stiffness–CMOD curves in Figure 10e are compared for the validation of the capabilities of the presented numerical model.

## 4. Discussion

Steel fibres significantly influence the behaviour of the otherwise very brittle HPC. While pure HPC fails after minimal crack openings, specimens with higher fibre contents can still transfer forces exceeding the initial crack load significantly, even at crack openings of more than 3 mm. As shown in Figure 5 and Figure 7, the plastic CMOD, residual stiffness and damage energy are suitable indicators to not only describe the deterioration process but also to differentiate between different fibre contents. Especially, the progression of the residual stiffness shows significantly higher values and a smoother transition to the opening of the macro-crack, especially in the crack-initiation phase from a CMOD of 0.02 mm for high fibre contents. The influence of the fibre quantity is also evident in the progress of the plastic fractions of the crack openings as well as the consumed energy. The results and development of damage indicators displayed comply well with studies in which similar loading–unloading schemes were used. For example, de Smedt [22] shows similar damage evolutions for two different types and lesser contents of hooked-end steel fibres in normal strength concrete. The tests performed here, therefore, show a generalizability to greater fibre contents and higher compressive strengths of the concrete. Results published by Boulekbache et al. [23] regarding residual stiffness and consumed energy are confirmed. The trends for all mentioned damage indicators as well as fibre content are comparable. Their results furthermore show positive effects of an increasing aspect ratio of the fibres as well as compressive strength of the concrete. However, the absolute values presented here are not directly comparable to Boulekbache et al. [23], since four-point bending tests are used there, resulting in multiple cracks in the tensile zone. In contrast, in the tests presented here, a single primary crack is induced in the center of the beam due to the weakened cross-section.

Nevertheless, scattering of the damage indicators is noticeable. However, it requires further investigation as to whether this is directly attributable to the amount of fibres, e.g., due to a more inhomogeneous fibre distribution resulting from comparatively poorer fresh concrete properties with increasing fibre quantities. The analysis of the acoustic emission provides additional insights into the damage behaviour. The comparison between the development of number of hits to the development of the energy of the acoustic events seems suitable to make the phases of crack initiation and macro-crack opening differentiable. The crack initiation is characterized by high numbers of hits with low energy content, whereas macro-crack opening is characterized by high energies at those acoustic events. This distinction between a crack-initiation phase and a crack-opening phase has first been introduced by Schorn [52] and Kopp [53]. De Smedt [22] reports that the evolution of acoustic emission can be used as alternative damage indicator. This finding can be supported here.

A phenomenological material model incorporating phase-field approach for fracture representing the damage behaviour of reinforced HPC at low cycle is presented. To describe the nonlinear features of HPC the Drucker–Prager plasticity model is formulated for the HPC phase. Additionally, a one-dimensional von Mises plasticity model is used to capture the non-linearity along preferred fibre direction. The Drucker–Prager plasticity model for the HPC phase, combined with different values of the critical fracture energy in the phase–field model, empower the presented model to calibrate the different parameters for concrete in tension and compression. The comparison of experimental and numerical results of three-point bending beam tests at low cycle shows the efficiency of the presented model. It is able to reproduce the main characteristics of the experimental results using measured mechanical properties and calibrated material parameters. For the validation, the degradation of the residual stiffness calculated using the numerical results and experimental data are compared. It can be observed that the model is not only capable of predicting the load-CMOD curves in terms of the load envelop, but also to efficiently predict the deterioration development in terms of residual stiffness and plastic CMOD during unloading-reloading cycles.

## 5. Conclusions

In this contribution a combined experimental–numerical approach is outlined aiming at the assessment of the high-cycle performance of steel fibre-reinforced HPC and UHPC. As a first step, low-cycle flexural fatigue tests on concrete with different fibre contents are conducted and different damage indicators, including acoustic emission analysis, are derived. Simultaneously, a phase-field approach for the fracture of steel fibre-reinforced HPC is presented.

From the experimental work, it is found that the evolution of stiffness and the plastic fraction of crack-mouth opening are damage indicators that show clear characteristics depending on the fibre content and are suitable for the calibration of numerical models. The absorbed damage energy is highly selective with respect to different fibre contents, but its applicability for calibration is numerically complex. The acoustic emission method allows the differentiation of different fracture processes, such as crack initiation and crack opening.

Furthermore it is shown, that the phenomenological material model incorporating a phase field approach for fracture in the pure HPC can be extended to predict fracture in steel fibre-reinforced HPC. The main feature is that, in contrast to incorporating the real spatial fibre distribution, the formulation using a structural tensor based on effective overall fibre distributions reduces the effort of their consideration in finite element discretization. This enables the feasible study of the influence of the different orientations and distribution of the embedded fibres on the overall material behaviour of HPC, e.g., load-CMOD curves. Additionally, the material model is not only capable of predicting the load-CMOD curve but also to predict damage evolution in terms the residual stiffness and the plastic CMOD. To study the capabilities, the presented model will be further developed for higher fibre contents that provide supercritical behaviour. Likewise, the modeling of higher cycle numbers is in progress and a comparison of damage development in low-cycle and high-cycle fatigue will be the future steps. Initial results, see [41], promise that the damage evolution is comparable.

## Figures and Tables

**Table 1:** Composition of the used concrete mixtures, data from [34].

Ingredients	Quantity in kg/m${}^{3}$, HPC
cement: CEM I 52,5R	500
fine sand	75
sand 0/2	850
basalt 2/5	350
basalt 5/8	570
silica fume	570
superplasticizer	5
stabilizer	3
water	176
steel fibres	0/23/57/115
number of specimens	3/3/3/3

**Table 2:** Mechanical properties of HPC, adapted from [34], compare [44,45].

Property	Mean Value	Unit
tensile strength $f_{t}$	5.7	MPa
compressive strength $f_{c}$	112	MPa
Young’s modulus *E*	39.976	GPa
Poisson’s ratio μ	0.192	–

**Table 3:** Settings for the measurements of acoustic emission.

Parameter	Value
duration discrimination time	0.4 ms
rearm time	0.4 ms
threshold	30.1 dB
filter	95 kHz–850 kHz
gain	34 dB

**Table 4:** Measured mechanical properties, see Table 2, and calibrated material parameters for HPC.

	$E^{\mathrm{HPC}}$	$\nu^{\mathrm{HPC}}$	$f_{t}$	$f_{c}$	$\psi_{t}^{c,\mathrm{HPC}}$	$\psi_{c}^{c,\mathrm{HPC}}$	$y_{0}^{\mathrm{HPC}}$	$\beta_{p}$	$\beta_{n}$	$h^{\mathrm{HPC}}$	*l*	ζ	$E^{F}$	$v^{F}$	$y_{0}^{F}$	$h^{F}$
	GPa	−	MPa	MPa	MPa	MPa	−	−	−	MPa	mm	−	GPa	−	MPa	MPa
HPC	39.976	0.192	5.7	112	4.2 $\times 10^{-4}$	0.13	6.2	0.5	0.12	13,000	14	1	210	0.003	660	130

**Table 5:** Calibrated values for the interpolation of degradation functions for HPC in tension and compression.

*q*	0	0.1	0.2	0.3	0.4	0.5	0.6	0.7	0.8	0.9	1
$g^{+}\left(q\right)$	1	1	1	1	0.93	0.85	0.74	0.6	0.39	0.18	0.003
$g^{-}\left(q\right)$	1	0.852	0.714	0.585	0.464	0.353	0.252	0.164	0.089	0.03	0.003

## Data Availability

Not applicable.
